# Anxiety about Mathematics and Reading in Preadolescents Is Domain-Specific

**DOI:** 10.3390/jintelligence12020014

**Published:** 2024-01-29

**Authors:** Delphine Sasanguie, Charlotte Larmuseau, Fien Depaepe, Brenda R. J. Jansen

**Affiliations:** 1 Research Centre for Learning in Diversity, HOGENT, 9000 Ghent, Belgium; delphine.sasanguie@hogent.be; 2 Faculty of Psychology and Educational Sciences@Kulak, KU Leuven, 8500 Kortrijk, Belgium; charlotte.larmuseau@howest.be (C.L.); fien.depaepe@kuleuven.be (F.D.); 3 ITEC, IMEC Research Group, KU Leuven, 8500 Kortrijk, Belgium; 4 Department of Psychology, Faculty of Social and Behavioural Sciences, University of Amsterdam, 1018 WS Amsterdam, The Netherlands

**Keywords:** math anxiety, reading anxiety, test anxiety, math performance

## Abstract

It was investigated whether test anxiety (TA), mathematics anxiety (MA), and reading anxiety (RA) can be traced back to some type of general academic anxiety or whether these are separable. A total of 776 fifth graders (*M_age_* = 10.9 years) completed questionnaires on TA, MA, and RA, as well as a mathematics test. Also, mathematics and reading performance results from the National Tracking System were requested. The sample was randomly split into two halves. Confirmatory factor analyses showed that a three-factor model (factors: TA, MA, RA) had superior model fit compared with a one-factor model (factor: “Academic anxiety”), in both halves. The resulting anxiety factors were related to math performance measures using structural equation models. A scarcity of data on reading performance prevented the analysis of links between anxiety and reading performance. Anxiety–math performance relations were stronger for MA than for TA and MA. We concluded that TA, MA, and RA are separable constructs.

## 1. Introduction

Learning mathematics and reading is accompanied by anxiety for some children (Hopko et al. 2001; Zbornik and Wallbrown 1991). In a past study, Hopko et al. (2001) categorized such academic anxiety as performance-based anxiety. These authors stated that performance-based anxiety occurs in (anticipation of) situations in which performance is expected and there is a risk of negative evaluation (Hopko et al. 2001). The combination of an academic setting and fear of negative evaluation separates academic anxiety from other anxieties or phobias. However, even to date, there is an ongoing debate on whether academic anxieties are general or domain-specific (e.g., Daker et al. 2022). Indeed, fear of negative evaluation may occur in all academic subjects because academics requires performance, which is tested regularly and, therefore, is intrinsically linked to (potential negative) evaluation. However, academic anxieties may also be rather domain-specific because an individual may only fear failing in a particular subject. In any case, the empirical evidence so far is inconclusive because some studies support the idea that math anxiety (MA) is distinct from test anxiety (TA) (e.g., Orbach et al. 2019), whereas other studies suggest that TA and MA are related (e.g., Punaro and Reeve 2012). Studies testing the domain specificity of reading anxiety (RA) are to date still scarce.

To counter feelings of academic anxiety, it is essential to determine if interventions should focus on academic anxiety in general, anxiety in a specific domain, or fear of failure in test situations (i.e., TA). If academic anxiety is more general, signals in one domain (e.g., math) may be indicative of anxiety in other school domains, as well, which would require a more general approach than when anxiety is tied to a certain domain only. In this study, we will first define academic anxieties and their relation to academic performance. Second, the evidence for and against the distinctiveness of academic anxieties is discussed, which results in the setup of the current study, in which we investigated whether anxieties regarding mathematics and reading and TA can be traced back to a general academic anxiety construct or are separable entities.

### 1.1. Academic Anxieties: Definitions and Relation to Academic Performance

TA is the negative emotion that occurs when academic performance is evaluated, or when anticipating such a situation. Highly test-anxious persons experience feelings of anxiety and physiological arousal (e.g., headaches, rapid breathing), worry about whether their performance will be good enough, and often try to avoid test situations (Zeidner 1998). Anxiety may deteriorate performance: affective and physiological symptoms cause distraction, worries occupy cognitive capacity (Eysenck and Calvo 1992), and avoidance disturbs one’s focus (e.g., rushing through a test to escape it) and practice. Indeed, children who report more TA typically perform lower on tests (although a U-shaped relation has also been proposed; McDonald 2001). On the other hand, low performance, such as failures in the past, may cause and increase TA (Zeidner 1998).

TA is observed in both primary and secondary schools (McDonald 2001). Its prevalence differs between grades and might be related to whether high-stakes testing takes place in a grade. In many countries, high-stakes testing takes place in the last compulsory school year, but students’ age in that school year differs per country (Putwain 2007).

Definitions of MA share the idea that people can experience symptoms of anxiety in an evaluative academic situation but then when being confronted with numbers, mathe-matical problems, or more general math-related topics. MA is characterized by negative emotional reactions (apprehension, tension), a general state of discomfort (Ashcraft and Ridley 2005; Ma and Xu 2004; Richardson and Suinn 1972), physiological arousal (Suárez-Pellicioni et al. 2016), worries (Ashcraft and Ridley 2005), and behavioral symptoms such as avoidance (Hembree 1990). As in TA, MA and math performance can influence one another in a vicious cycle, and the relation between the two constructs is, thus, most probably reciprocal (Carey et al. 2016). Self-reports of MA and math performance indeed correlate negatively (*r* ≈ −0.3) (Ashcraft and Moore 2009; Barroso et al. 2021; Chang and Beilock 2016; Dowker et al. 2016; Namkung et al. 2019; Suárez-Pellicioni et al. 2016; Zhang et al. 2019).

The reported prevalence of MA varies between 2 and 6% (Chinn 2009) to as much as 68% (Betz 1978) of the population, depending on the cutoff criteria, measures, and population of interest. MA can arise in educational/academic or professional contexts but also in ordinary life situations, for instance, when paying for groceries (Ashcraft and Moore 2009). People who report more MA are more likely to avoid activities and situations in which math skills are needed, including math-related courses and certain careers like science, technology, or engineering (Ashcraft and Ridley 2005; Hembree 1990; Ma and Xu 2004; Maloney and Beilock 2012). Therefore, MA might not only directly affect the development of math skills, but might also have other, far-reaching consequences. Indeed, highly math-anxious persons often have higher health costs (Duncan et al. 2007; Parsons and Bynner 2005; Reyna et al. 2009; Woloshin et al. 2001), lower socioeconomic statuses (Ritchie and Bates 2013), and mortgage defaults (Gerardi et al. 2013).

Like TA and MA, RA is characterized by affective, physiological, cognitive, and avoidance symptoms in an evaluative academic context that also occurs in situations that require reading (e.g., reading aloud in the classroom), both in the actual moment and in anticipation of it (Jalongo and Hirsh 2010; Piccolo et al. 2017; Zbornik and Wallbrown 1991). The risk that reading motivation and involvement in reading may decay over time as a result of RA (Piccolo et al. 2017; Zbornik 1988) underlines its importance. RA has mostly been studied in the context of foreign language learning (Horwitz 1986). Only a few studies link RA to native language (Jalongo and Hirsh 2010; Piccolo et al. 2017).

Also, for RA, the anxiety can just as well be a cause of low performance because it charges working memory, leaving less cognitive capacity available for reading (Katzir et al. 2018). It can also be the result of early failures through negative consequences, such as humiliation and negative feedback (Jalongo and Hirsh 2010; Katrancı and Kuşdemir 2016)^1^. Indeed, a negative, significant relation between RA and reading performance has been observed (*r* ≈ −0.28; Katzir et al. 2018; *r* ≈ −0.4; Pollack et al. 2021; *r* = −0.29; Ramirez et al. 2019; *r* ≈ −0.4; Zbornik and Wallbrown 1991). 

### 1.2. Separate Entities or a General Academic Anxiety Construct?

In sum, TA, MA, and RA share a fear of negative evaluation in an academic context, as well as the anxiety symptoms of affect, physiology, cognition, and avoidance. Earlier studies have already investigated whether TA and MA are part of a general academic anxiety construct or separate anxieties (Dew and Galassi 1983). Here, we recapitulate and slightly complement the excellent review by Dowker et al. (2016) and the existent findings on whether MA can be considered as an entity on its own. First, MA has a relatively strong correlation with TA in children (*r* = 0.71; Carey et al. 2017), preadolescents (*r* = 0.69; Carey et al. 2017), and adults (*r* between 0.3 and 0.5 in Hopko et al. (1998) and Hembree (1990); *r* between 0.36 and 0.80 in Kazelskis et al. (2000)). Second, a somewhat smaller, but consistent, relationship was reported between MA and general anxiety for children, preadolescents, and adults (*r* = 0.29 in Grade 2 and *r* = 0.38 in Grade 3 in Cargnelutti et al. (2017); *r* = 0.35 in adults, Hembree (1990); mean *r* = 0.42 for children and mean *r* = 0.39 for preadolescents in Hill et al. (2016)). A weaker relation between MA and general anxiety than between MA and TA would make sense because the latter are both rooted in experiences of school and performance, whereas general anxiety serves as a background variable explaining some of the shared variance between MA and TA (Carey et al. 2017; Dowker et al. 2016). Third, a confirmatory factor analysis of trait MA (stable anxiety across math-related situations), state MA (temporary experience of anxiety in specific math-related situations), TA, and social anxiety questionnaire scores showed that the best model included separate MA factors (one for trait MA and one for state MA). Also, only the MA factors were related to math performance, not the combined factor of social anxiety and TA (Orbach et al. 2019). Finally, previous studies have also described anxiety toward academic subjects other than mathematics: anxiety about learning, reading, and use of (foreign) language (Carroll et al. 2005; Carroll and Iles 2006; Cheng et al. 1999; Ghonsooly and Loghmani 2012; Horwitz 1986; Wu and Lin 2014), music performance anxiety (Kenny 2011), and anxiety and lack of confidence in drawing (e.g., Cox 1989; Golomb 2002; Thomas and Silk 1990).

Studies directly comparing anxiety about different academic subjects in one and the same sample are scarce. Punaro and Reeve (2012) measured the MA and literacy anxiety of 9-year-old Australian children and related their anxiety and actual academic abilities. Children showed more anxiety toward mathematics than literacy. Moreover, latent profile analyses per domain identified a three-cluster solution of low, moderate, and high anxious subgroups twice. The relationship among the three subgroups across the math and language domains was significant, suggesting that there is a tendency to belong to the same anxiety subgroup in the math and literacy domains. The high-anxious math subgroup displayed poorer math performance than the other math subgroups, demonstrating a link between MA and math performance. No relationship was found between any of the literacy anxiety subgroups and literacy performance. In sum, the results by Punaro and Reeve seem to suggest that although children’s worries about mathematics were greater than for literacy, MA (at least partly) overlaps with literacy anxiety. Recently, Pollack et al. (2021) assessed anxiety, motivation, and performance in both math and reading in a sample of 8- to 13-year-olds in the U.S. and observed that MA and RA were positively related. Although anxiety and performance measures were correlated within each domain, there were cross-relations, as well, with RA predicting math performance; however, MA did not predict reading performance. Finally, Carey et al. (2017) measured MA, TA, general anxiety, and mathematics and reading performance in large samples of 8- to 9-year-olds and 11- to 13-year-olds. Latent profile analyses revealed a subgroup of individuals exhibiting higher MA and TA but lower general anxiety, which was interpreted as the “Academic anxiety” profile. Moreover, next to the expected relation between MA and math performance in the collapsed sample (*r* = −0.29), they also observed a correlation of −0.17 between MA and reading performance. The authors proposed that there were children who developed MA as a result of poor math performance, because math and reading performance are often closely related (e.g., *r* = 0.73 in this study), or that children with MA are more likely to have other forms of “Academic anxiety”, like RA, which impact their reading performance. However, as the authors recognize themselves, it is impossible to judge whether one or both of these mechanisms are at play because RA was not measured in that study.

Studies on whether RA can be considered a separate construct are rare. Zbornik and Wallbrown (1991) assessed children’s RA, general anxiety, math and reading performance, and grades in spelling, reading, and math. The negative, significant correlations between RA scores and reading performance (*r* ≈ −0.4) appeared stronger than those between general anxiety scores and reading performance (*r* ≈ −0.2) and higher than those between RA scores and math test performance (*r* = −0.34) and math grades (*r* = −0.2). Finally, the correlation between RA scores and general anxiety scores was positive, *r* = 0.46.

Overall, the existent evidence with regard to the question of whether TA, MA, and RA can best be considered as aspects of a general academic anxiety or as separate constructs is scarce and unsatisfying. In favor of the hypothesis that TA, MA, and RA are aspects of a general academic anxiety are the findings that the relations between TA, MA, and RA are positive and significant (Dowker et al. 2016; Zbornik and Wallbrown 1991), a profile analysis shows clusters of children with comparable levels of general anxiety, TA, and MA (Carey et al. 2017), the profiles across domains are dependent (Punaro and Reeve 2012), and anxiety scores and performance across domains show significant, negative correlations (Carey et al. 2017; Pollack et al. 2021; Zbornik and Wallbrown 1991). In favor of the alternative hypothesis that TA, MA, and RA are separable are the moderate relations between MA and RA on the one hand and TA and general anxiety on the other hand (Dowker et al. 2016; Zbornik and Wallbrown 1991), the separate factors for MA and TA in a factor model (Orbach et al. 2019), and the findings that anxiety–performance relations seem stronger within domains than they do across domains (Carey et al. 2017; Pollack et al. 2021; Zbornik and Wallbrown 1991).

### 1.3. Current Study

To study whether the anxieties are aspects of general academic anxiety or are separable constructs, we measured TA, MA, and RA, as well as the math and reading performance of 776 preadolescents (i.e., fifth graders, ±11 years old). We focused on this age group to be able to compare and extend the results of the most relevant studies to date in this regard (Carey et al. 2017; Pollack et al. 2021; Punaro and Reeve 2012). To measure TA, a subtest from the Dutch questionnaire “SchoolVragenLijst” (Smits and Vorst 2008) was used. For MA and RA, two new instruments were introduced for the country in which this study was conducted: a direct translation of the revised Child Math Anxiety Questionnaire (CMAQ-R; Ramirez et al. 2016) and an adaptation of this questionnaire to the reading context. For math performance, a speed test assessing skills in basic math was administered (De Vos 1994). Results from the mathematics and reading sections of the National Tracking System were requested as indicators of both math and reading performance.

Using confirmatory factor analysis (CFA), we contrasted a one-factor model (academic anxiety) versus a three-factor model (separate anxieties: TA, MA, and RA). However, responses to each anxiety questionnaire can possibly stem from multiple underlying constructs. First, feelings may be different in a test context than when working on academic tasks without anything being at stake. Whereas the TA questionnaire exclusively asks about the test context, questions in the MA and RA questionnaires either refer to a test context or to a context in which the student performs a math or reading task without being tested. Second, anxiety reports may be impacted by the specific anxiety component that is being queried. Whereas items of the TA scale concern either affect or cognition, the MA and RA questionnaires only concern affect. In S1, we elaborate on these alternative models in which context and anxiety components are taken into account.

In case the CFA showed that the three-factor model with separate anxiety factors fitted best, the anxiety factors were related to performance in each domain (math/reading), and the relations were compared between factors. The hypothesis of a general academic anxiety would be supported if the relations of all anxiety factors with performance in both domains were comparable, whereas the separability of TA, MA, and RA would be more likely if the relation between the anxiety factor and performance in the same domain was strongest.

## 2. Materials and Methods

### 2.1. Participants

A group of 776 fifth graders from 33 elementary schools in Flanders, Belgium, participated. Participating schools and children were recruited by 25 bachelor’s students in psychology and educational sciences for the purpose of this study, which was part of an assignment for a course. The students collected the data during February–May 2018. The school, as well as all parents and children signed a written informed consent letter. After data collection, data were anonymized in a function of data analysis. Ethics approval was obtained from the local ethics committee (G-2017 10 951).

The data of participants who were diagnosed with dyslexia (*N* = 7), dyscalculia (*N* = 2), or both (*N* = 1) and of those who followed special education for math (*N* = 7) were removed from further analysis. Next, a cutoff of 10% was used for missing data on the anxiety questionnaires (i.e., more than 1 question of a questionnaire), which are the main focus of the analyses. Two participants missed more than 1 MA item, and three participants missed more than 1 item on the RA questionnaire. Although scores outside the 95% confidence interval around the mean were demonstrated by 9 (MA) and 14 participants (RA), outliers were not excluded from further analysis because distributions from anxiety questionnaires are often skewed right (e.g., Carey et al. 2017). The final sample consisted of 754 participants, which was randomly split in two halves (both *N* = 377). In Sample A, the average age was 130.8 months (±11 years; *SD* = 4.56 months; missing age data for 7%), and 48.5% of the sample were boys. In Sample B, the average age was 130.7 months (*SD* = 4.66 months; missing age data for 10%), and 49.3% of the sample were boys.

### 2.2. Materials

#### 2.2.1. Anxiety Questionnaires

To measure TA, the subtest “Self-confidence during tests” from the Dutch questionnaire “SchoolVragenLijst” (SVL; Smits and Vorst 2008; see Appendix A for item descriptions) was used. Children were asked to indicate for each of 16 statements (e.g., “Right before a test, I am afraid that I will forget something because of feelings of tension”) whether the statements applied to them (response options: “that’s not true”—1 point, “I don’t know”—2 points, “that’s true”—3 points). Internal consistency in the current sample was good, *α* = 0.87.

To measure MA, the Dutch translation of the revised Child Math Anxiety Questionnaire (CMAQ-R; Ramirez et al. 2016; see Appendix A for the English items) was used. In each of the 16 items, children were asked to indicate how nervous they would feel in a math-related situation (e.g., “How do you feel when you look at your math book and you see all the numbers in it?”) by circling one of five smiley faces displaying an emotional gradient from not “nervous at all” (1) to “very, very nervous” (5) in left-to-right order. The original version of the CMAQ-R had good internal consistency (Ramirez et al. 2016). Internal consistency in the current sample was also good, *α* = 0.89.

To measure RA, the 16 items of the CMAQ-R (Ramirez et al. 2016; see Appendix A for the English items) were minimally adjusted to a reading context (e.g., “How do you feel when you take your reading book and see all the texts and words in it?”), using the same response scale. Hereafter, we will refer to this questionnaire as the Child Reading Anxiety Questionnaire (CRAQ). Internal consistency in the current sample was good, *α* = 0.92.

Higher scores on the anxiety questionnaires indicated higher anxiety. The mean score across completed items was used, but the mean was only calculated for a questionnaire if the participant missed none or only one item on the questionnaire. The Dutch translation of the CMAQ-R, and the parallel Dutch CRAQ can be obtained from the authors on request.

#### 2.2.2. Math and Reading Performance

The paper and pencil Tempo Test Rekenen (Tempo Test Math; TTR; De Vos 1994) was administered to measure math performance. The TTR consists of five columns of bare math problems (e.g., “1 + 1 =”) using the operations addition, subtraction, division, and multiplication and a mix of these operations. For each column, the participant is asked to solve as many problems as possible in one minute. A total of 718 participants completed the TTR correctly (some children made mistakes because they did not understand the instructions correctly). Sum scores across the five columns were used (possible range 0–200).

Parents/caretakers were asked for permission to request their children’s scores in the national tracking system (NTS; “LeerlingVolgSysteem”) from the school for grades 3 (Van Rompaey and Vandenberghe 2016) and 5 (Huybens et al. 2017). NTS data on math were available for 530 participants in grade 3 and 686 participants in grade 5. Considering reading, a majority of the schools did not administer the NTS standardized tests (i.e., these assessments are not compulsory). NTS data were requested for technical reading in grades 3 (Van Rompaey and Vandenberghe 2013) and 5 (Van Rompaey and Vandenberghe 2014), as well as for reading comprehension (Krom and Staphorsius 1998). However, the data were not included in the data analyses because schools only had data for a very limited number of children.

### 2.3. Procedure

Assessment took place in the classroom. The TTR was administered first following the manual’s instructions (De Vos 1994). Questionnaires were administered next in fixed order (i.e., MA, RA, TA). Test assistants (i.e., the bachelor’s students who administered this study as part of their assignment for the course “Developmental Psychology”) warned the participants not to think too long before answering and that there were no incorrect answers. On average, test administration took 45–50 min (i.e., one class hour in the Flemish school system).

### 2.4. Analytic Strategy

**Figure 1:**
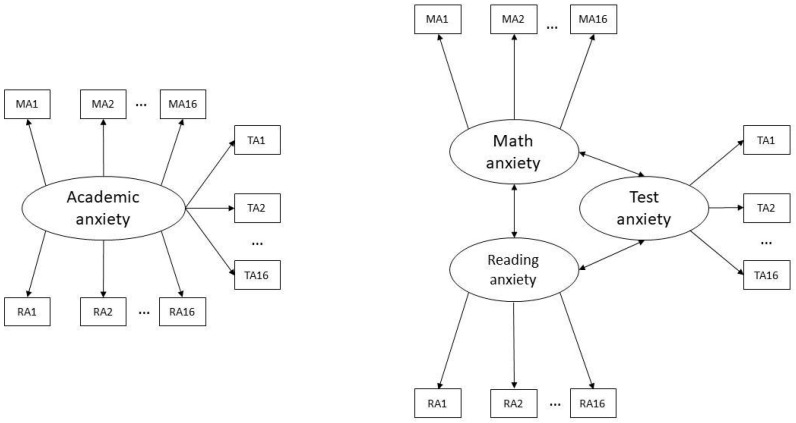
One-factor model (**left**) and three-factor model (**right**). TA, MA, RA = test anxiety, math anxiety, reading anxiety.

For the central research question, confirmatory factor analyses (CFA) were conducted using the Lavaan package in R (version 3.5.1; Rosseel 2012). The CFA models were first estimated for half of the sample (Sample A). To validate findings, prevent overfitting, and investigate the reliability and generalizability of the selected model, we re-ran the CFA’s on the other half (Sample B). The maximum likelihood parameter estimation (MLM-chi-square) with standard errors and a mean-adjusted chi-square test statistic (i.e., Satorra-Bentler *χ*^2^) was employed because the distributions of the mean scores on the anxiety questionnaires significantly deviated from a normal distribution in both samples. This estimation procedure has minimal demands on data assumptions and is robust to non-normality (Rosseel 2012). First, a single-factor model and a three-factor model (see Figure 1) were estimated for Sample A. In the single-factor model, general academic anxiety is the main latent variable with items of all anxiety questionnaires as indicators. In the three-factor model, TA, MA, and RA are latent variables, and each latent variable has its own observed indicators.

Second, the best-fitting model was selected by inspecting the fit indices *χ*^2^, normed *χ*^2^-test (*χ*^2^/*df*), comparative fit index (CFI), Tucker–Lewis index (TLI), root mean square error of approximation (RMSEA), and standardized root mean square residual (SRMR) (Brown 2015), as well as by testing whether the *χ*^2^-difference between models was significant. The normed *χ*^2^-test should have a value smaller than 2.0 (Kline 2013). Values of CFI and TLI between 0.90 and 0.95 would be acceptable, as well as a value < 0.06 for RMSEA and a value < 0.08 for SRMR (Hu and Bentler 1999).

Third, items with low standardized factor loadings were deleted to optimize model fit because size-of-factor loadings and model fit are related (Kline 2013; Niemand and Mai 2018; Sharma et al. 2005). Although the criteria for factor loadings are in use, setting thresholds remains arbitrary (Niemand and Mai 2018). Here, to balance model fit and retaining items, we first deleted items with factor loadings lower than 0.3, and if model fit was still unacceptable, also deleted items with factor loadings lower than 0.4. After cross-validating the results in Sample B, the samples were merged again, and the final model was estimated for the full sample.

Because of the large sample size, we were able to further investigate the reliability and validity of the instruments that had been translated into Dutch, in addition to the central research question. The composite reliability (CR) of the factor(s) in the selected measurement model was calculated in the full sample to further determine internal consistency. The average variance extracted (AVE) was calculated as an index of convergent validity. AVE is the amount of variance that is captured by a construct in relation to the amount of variance due to measurement error.

If the selected model had multiple factors, the anxiety factors were included as predictors of each math performance measure in a structural equation model (SEM) for the full sample. For each math performance measure, a separate SEM was estimated. The maximum likelihood parameter estimation with robust standard errors (MLR) was applied, using full information maximum likelihood to handle missing data. We were interested in the relative strength of within-domain relations (e.g., between the MA factor and a math performance measure) in comparison to cross-domain relations (e.g., between the RA factor and a math performance measure). Unfortunately, these analyses were not conducted for reading performance because of the scarcity of reading performance data.

## 3. Results

### 3.1. Descriptive Statistics

Table 1 shows the means and standard deviations of the anxiety questionnaires and performance measures, per sample. Please note that data on the performance measures were not available for all participants, as described in Section 2.2.2. Independent *t*-tests showed that the differences between samples were not significant.

Table 2 shows the Pearson correlations between the scores on the anxiety questionnaires and the various measures of math and reading performance for the full sample for which performance data were available (Samples A and B combined). As expected, MA scores and math performance measures correlated negatively and significantly, *r* = −0.29, *p* < .001 for TTR; *r* = −0.30, *p* < .001 for NTS math performance grade 3; *r* = −0.33, *p* < .001 for NTS math performance grade 5. Correlations between RA scores and reading performance measures were negative and around the alpha-level of 0.05, *r* = −0.22, *p* = .083 for technical reading performance in grade 3 and *r* = −0.30, *p* = .046 for technical reading performance in grade 5. Note that data on reading performance were only available for a small number of participants.

### 3.2. Latent Factor Models of Anxiety Measures

Table 3 shows the fit indices of the estimated factor models in Sample A. The one-factor model did not reach the thresholds for the normed *χ*^2^-test, CFI, TLI, RMSEA, and SRMR. For the three-factor model, thresholds were reached for the normed *χ*^2^-test, RMSEA, and SRMR but not for CFI and TLI. Studies have shown that CFI and TLI value larger than 0.90 are needed to ensure that miss-specified models are not accepted (Kline 2013). In the three-factor model, the latent factors were correlated (*r* = 0.64 for MA and RA; *r* = 0.62 for MA and TA; *r* = 0.53 for RA and TA). Generally, when factors are moderately to highly correlated, a general factor may underly the data. Nevertheless, based on acceptable to good values for most fit indices, the three-factor model was selected. Additionally, the *χ*^2^-difference test indicated that the three-factor model fitted the data significantly better than the single-factor model (*χ*^2^(3) = 924.6, *p* < .001). Next, items with poor factor loadings were systematically removed from the three-factor model. Model fit was inadequate after the deletion of items with factor loadings <0.3; however, it was adequate after the deletion of items with factor loadings <0.4. In this model, the construct of TA had 9 items, MA 9 items, and RA 15 items. Latent correlations were significant: *r* = 0.59 for MA and RA; *r* = 0.63 for MA and TA; and *r* = 0.54 for RA and TA. The Supplementary Materials show the results of fitting the alternative latent factor models to the data in Sample A. Again, a model in which the anxieties were considered separate had superior fit to models in which an overarching construct of academic anxiety was assumed.

**Figure 2:**
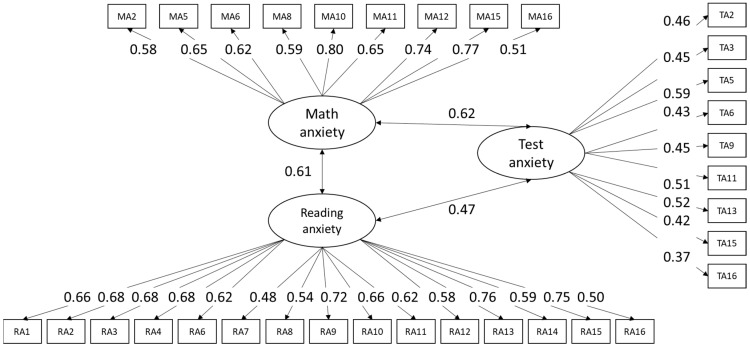
Standardized factor loadings and correlations between the factors of the three-factor model, full sample. TA, MA, RA = test anxiety, math anxiety, reading anxiety. All loadings and correlations are significant (*p* < .001).

The procedure above was repeated for Sample B. The same items that were deleted to optimize model fit in Sample A were deleted in the models for Sample B. Fit indices were comparable for the samples, and the three-factor model was also the best-fitting model for Sample B (Table 3), which strengthens the weight of proof that the three factors (i.e., TA, MA, RA) can be distinguished (Schreiber et al. 2006). Finally, the three-factor model was estimated for the re-merged total sample (see Table 3 for fit measures and Figure 2 for the estimates for the three-factor model). Although the normed *χ*^2^-test of the final model for the complete data was higher than two, all other fit measures reached criterion and were acceptable. The factor loadings in the final model were all significant and between 0.37 and 0.80.

Appendix A provides the items or item descriptions of the questionnaires, including means and factor loadings for those questions that were maintained in the selected model. It seems that all but one of the deleted MA items concerned a specific math problem. The other deleted MA item concerned a shopping situation, just like the deleted RA item. Many TA items that were deleted concerned situations following a test, whereas most maintained items concerned situations before and during test taking.

### 3.3. Psychometric Properties of Anxiety Factors in the Measurement Model

Table 4 shows composite reliability and average variance extracted by anxiety factor in the selected model. CR exceeded the threshold of 0.70. The AVE of the three constructs did not reach the threshold of 0.50. However, given that CR is higher than 0.70, we can accept a threshold for AVE of 0.40 (Fornell and Larcker 1981), which was reached by the CMAQ-R and CRAQ.

### 3.4. Anxiety–Performance Relations

Table 5 shows that most fit indices of the SEMs for the math performance measures were acceptable. Table 6 shows the estimated standardized coefficients concerning the relations between each anxiety factor and the math performance measures. The estimates of the relations between the MA factor and each math performance measure were negative and significant, whereas those concerning the relation with the RA factor were nonsignificant. In addition, the relation between the TA factor and each math performance measure was negative and significant.

## 4. Discussion

Fifth graders completed questionnaires on test anxiety (TA), math anxiety (MA), and reading anxiety (RA) to investigate whether TA, MA, and RA can be traced back to a general academic anxiety or whether they are separable. Cross-validated confirmatory factor analyses showed that the model with three anxiety factors showed a better fit than the model with one general factor. Next, factors were related to math performance. Both the TA and MA factors were negatively related to math performance, whereas the RA factor was not. The results of a recent study by Daker et al. (2022) suggest, similarly, that the performance between anxiety and performance is “cognition-specific”. These authors showed that anxiety in a given cognitive domain (i.e., math) predicted differences in performance within that domain, even when controlling for general trait anxiety and anxiety in a closely related cognitive domain (i.e., spatial reasoning).

The results of the current study suggest that TA, MA, and RA should be considered as separable constructs, confirming earlier results (Hembree 1990) but also extending them because the current results are based on a cross-validated measurement model and a younger sample. This finding suggests that interventions aimed at separate school subjects can be more beneficial than a general intervention aimed at academic anxiety. However, the correlations between the anxieties are considerable, implying that one should be alert to anxiety in multiple subjects when anxiety is observed in a particular school subject.

In optimizing the model fit of the measurement model, some items were deleted. A similarity of most deleted items of the CMAQ-R is that they concern a specific math problem. An inspection of the math problems in the deleted items gave the impression that these were easier than those in the undeleted items, which was supported by lower reports of anxiety on the deleted items compared with the undeleted items. Substitution of these problems with more difficult math problems in a future version may further improve internal consistency of the CMAQ-R and prevent item deletion. Alternatively, using more conceptual descriptions of math problems (e.g., “How would you feel if you had to solve a difficult multiplication problem?”) can possibly decrease both the risk of item deletion and the dependency of the CMAQ-R on students’ math ability. Also, many items on the TA subtest of the established SVL (Smits and Vorst 2008) were removed because factor loadings were low. It seems that undeleted items concern situations prior to and during a test, whereas deleted items concern situations following a test, such as anticipating test results. Possibly, the TA construct that is measured with the remaining items is slightly more limited than when using the full scale and mainly concerns anxiety in test situations and anticipating test situations but not anticipating test results.

Although TA, MA, and RA are best conceived as distinct constructs, they are not independent because they correlated moderately positively in the current study, as well as in earlier studies (see Dowker et al. 2016 for an overview; Carey et al. 2017; Pollack et al. 2021; Zbornik and Wallbrown 1991). That is, children who reported anxiety in one school subject were more likely to report anxiety in another subject and higher TA. These correlations do not seem to stem from general poor school performance (Carey et al. 2017) because it was also observed that MA, but not RA, was related to math performance, which suggests that anxiety–performance relations across school subjects are weaker than those that concern the same school subject. TA, MA, and RA can also not be considered independent from general anxiety (Hembree 1990; Hill et al. 2016; Zbornik and Wallbrown 1991). A future study including TA, MA, RA, as well as general anxiety, can reveal the interdependence and provide additional information on the target of academic anxiety treatments.

Because the CMAQ-R and CRAQ were newly developed instruments or new for the country in which this study took place, the psychometric properties of the questionnaires were investigated. The Dutch translation of the CMAQ-R (Ramirez et al. 2016) was internally consistent, both as a full scale and after item deletion in favor of model fit. Convergent validity was sufficient and the relation with math performance was similar to that often found in the literature, supporting criterion validity. The adaptation of the CMAQ-R to the reading context, resulting in the CRAQ, also showed good internal consistency (both for the full scale and after item deletion for the benefit of model fit) and sufficient convergent validity. The relation between RA and reading performance can be studied in future research. We conclude that both the Dutch CMAQ-R and the CRAQ appear to be reliable and demonstrate sufficient convergent validity, as well as that the Dutch CMAQ-R also shows acceptable criterion validity but that further research into criterion validity is required for the CRAQ.

In the current study, children reported similar levels of MA and RA, whereas children in the study by Punaro and Reeve (2012) reported higher levels of MA than literacy anxiety. A possible explanation is that literacy anxiety (study by Punaro and Reeve) concerns a broader concept than reading, also including word knowledge, for example, than RA (this study). Other explanations are international differences in curricula and views on the difficulty of math and language. Also, the children in the study by Punaro and Reeve were 9-year-olds, whereas the children in the current study were about 11 years old.

To study the research question of whether academic anxieties can be considered separate or can be traced back to a general academic anxiety, we chose a parsimonious method by only fitting a one-factor model and a three-factor model. Both the hierarchical model and the bifactor model seem to be interesting alternative models because both allow for combining a general factor and specific factors. These models were, however, not fitted for theoretical and methodological reasons. A hierarchical model for the current data would include a higher-order factor influencing three underlying factors. However, the model would be equivalent to the three-factor model in which the relations between the underlying factors would be constrained to zero and result in a saturated model in which the relations would be estimated freely. A bifactor model for the current data would include a general factor and three unrelated specific factors. However, fit indices may be biased to the bifactor model; that is, fit indices can favor the bifactor model, even when data are simulated with a correlated-factors model (Greene et al. 2019). Also, interpretation of the specific factors would be complicated for the current data (Dolan and Borsboom 2023). Each item would load on the general factor and a specific factor. Whereas the general factor may be interpreted as (academic) anxiety, the interpretation of the specific factors is unclear. Interpretation in terms of anxiety seems implausible because the specific factors are not related to the general factor. However, interpreting the specific factors in terms of constructs unrelated to anxiety also seems implausible because the common denominator in items loading on the same factor is test anxiety or domain-specific anxiety.

A first important limitation of the current study is the limited availability of reading performance data. No reading test was administered because there was insufficient time for individual assessment. Moreover, not all participating schools used the reading tests of the National Tracking System. Therefore, we decided not to include the reading performance data in the analyses. In future research, reading performance data are required to test the relations with the various anxiety forms. A second limitation is the use of self-report questionnaires to assess anxiety. The advantages of questionnaires are their scope (a wide range of situations can be presented in a short time using descriptions), reliability, and ease of (group) data collection. However, questionnaires have disadvantages, as well, especially in use with children because questionnaires require the capacities of vocabulary, memory, and introspection and increase the risk of biases, such as social desirability, and the misinterpretation of questions. More recently, other measures, such as heart rate, skin conductance, cortisol secretion, brain imaging (for an overview, see Dowker et al. 2016), and cognitive bias tasks, have been applied to study MA (Schmitz et al. 2019). Future studies may combine questionnaires and such alternative measures.

A third limitation is that the order of the administration of questionnaires was fixed. The RA questionnaire preceded the TA questionnaire, and this may have primed participants to respond to the TA items with reading experiences in mind. A randomized order of questionnaires is preferred in future work. A fourth limitation is the participants’ restricted age range. Conducting this study in other age groups could shed light on the development of anxiety in each domain. The literature on the development of MA is, for example, not conclusive. Such studies may also address the question of whether the domain-specificity of anxieties develops over time. Possibly, children learn more and more about their specific strengths and weaknesses; however, it is also possible that anxiety about a specific school subject generalizes to a general fear of failure in academics.

To conclude, the present study supported the hypothesis that TA, MA, and RA are separable constructs by applying and cross-validating confirmatory factor models to the responses of a large sample of children around the age of 11 years. The resulting MA factor, but not the RA factor, is specifically related to math performance. Also, this study introduced the translation of the original English CMAQ-R (Ramirez et al. 2016) to a Dutch-speaking country and supported its internal consistency, as well as its convergent and criterion validity. Finally, this study adapted the CMAQ-R to a reading context and demonstrated that this new questionnaire, CRAQ, was internally consistent and had sufficient convergent validity. Although further research is required before the questionnaires can be applied in individual diagnostic assessment, the current study has taken important steps in the study of the meaning of TA, MA, and RA for children’s academic careers.

## Figures and Tables

**Table 1:** Means (standard deviations) of anxiety and performance measures, compared between Samples A and B.

	Sample A (*N* = 377)	Sample B (*N* = 377)	*p* (*t*-Test)
Instruments administered in this study			
Math anxiety (Dutch CMAQ-R, range 1–5)	1.77 (0.55)	1.81 (0.56)	.323
Reading anxiety (CRAQ, range 1–5)	1.79 (0.63)	1.86 (0.66)	.123
Test anxiety (SVL, range 1–3)	1.87 (0.45)	1.89 (0.45)	.476
Math performance (TTR, range 0–200)	115.32 (21.23)	114.18 (21.77)	.479
	(*N* = 361)	(*N* = 357)	
Instruments from the national tracking system			
Math performance, grade 3 (range 0–63)	45.01 (9.44)	45.09 (9.62)	.919
	(*N* = 279)	(*N* = 251)	
Math performance, grade 5 (range 0–61)	43.05 (10.08)	43.22 (10.40)	.823
	(*N* = 351)	(*N* = 335)	
Technical reading performance, grade 3 (0–120)	70.18 (20.45)	73.19 (14.65)	.494
	(*N* = 34)	(*N* = 31)	
Technical reading performance, grade 5 (0–120)	85.50 (24.95)	86.45 (19.17)	.887
	(*N* = 24)	(*N* = 20)	

**Table 2:** Pearson correlations (*N* in parentheses) between anxieties and performance in mathematics and reading.

	2.	3.	4.	5.	6.	7.	8.
1. Math anxiety (Dutch CMAQ-R)	0.60 ** (754)	0.53 ** (754)	−0.29 ** (718)	−0.30 ** (530)	−0.33 ** (686)	−0.05 (65)	0.02 (44)
2. Reading anxiety (CRAQ)		0.43 ** (754)	−0.17 ** (718)	−0.19 ** (530)	−0.21 ** (686)	−0.22 (65)	−0.30 * (44)
3. Test anxiety (SVL)			−0.25 ** (718)	−0.26 ** (530)	−0.33 ** (686)	−0.10 (65)	−0.12 (44)
4. Math performance (TTR)				0.41 ** (528)	0.47 ** (684)	0.38 ** (65)	0.55 ** (44)
5. Math performance, grade 3					0.71 ** (526)	0.10 (65)	−0.06 (43)
6. Math performance, grade 5						0.21 (64)	0.15 (44)
7. Technical reading perf., grade 3							0.73 ** (43)
8. Technical reading perf., grade 5							

Note. * *p* < .05, ** *p* < .01.

**Table 3:** Fit indices for the latent factor models of the anxiety questionnaires.

Model	*χ* ^2^	*df*	*χ*^2^/*df*	RMSEA	SRMR	CFI	TLI
Sample A							
1-factor	2895.00	1080	2.68	0.08	0.09	0.64	0.63
3-factor	1970.35	1077	1.83	0.05	0.06	0.83	0.82
3-factor, items deleted with loadings ≤ 0.3 *^a^*	1849.80	986	1.88	0.06	0.06	0.83	0.83
3-factor, items deleted with loadings ≤ 0.4 *^a^*	847.94	492	1.72	0.05	0.05	0.91	0.90
Sample B							
1-factor	3133.18	1080	2.90	0.08	0.09	0.60	0.59
3-factor	1910.92	1077	1.77	0.05	0.06	0.84	0.83
3-factor, items deleted with loadings ≤ 0.3 *^a^*	1755.63	986	1.78	0.05	0.06	0.85	0.84
3-factor, items deleted with loadings ≤ 0.4 *^a^*	821.59	492	1.67	0.05	0.06	0.91	0.90
Total Sample							
3-factor, items deleted with loadings ≤ 0.4 *^a^*	1111.68	492	2.26	0.05	0.05	0.92	0.91

Note. *^a^* Items with factor loadings ≤ 0.3/0.4 in the previously estimated model were deleted.

**Table 4:** Measures of reliability and convergent validity of anxiety factors in revised three-factor model.

	Composite Reliability	Average Variance Extracted
Test anxiety	0.82	0.34
Math anxiety	0.87	0.43
Reading anxiety	0.91	0.42

**Table 5:** Fit indices for structural equation models linking anxiety factors to math performance measures.

*N*	Dependent Variable	*χ* ^2^	*df*	*χ*^2^/*df*	RMSEA	SRMR	CFI	TLI
713	TTR	1238.49	522	2.37	0.05	0.04	0.91	0.90
525	NTS: Math, grade 3	1198.33	522	2.30	0.05	0.04	0.91	0.91
681	NTS: Math, grade 5	1193.34	522	2.29	0.04	0.04	0.91	0.91

**Table 6:** Estimates of standardized coefficients of the relations between anxiety factors and math performance measures in the structural equation models.

		Anxiety Factor		
*N*	Dependent Variable	Math Anxiety	Reading Anxiety	Test Anxiety
713	TTR	−0.24 **	0.04	−0.14 *
525	NTS: Math, grade 3	−0.24 **	0.02	−0.15 *
681	NTS: Math, grade 5	−0.24 ***	0.04	−0.22 ***

Note. * *p* < .05; ** *p* < .01; *** *p* < .001. See Appendix B for SEM plots.

## Data Availability

The data presented in this study will be made available after acceptance on the Open Science Framework repository.

## Note

1 A Turkish study on the relation between RA and reading performance Katrancı and Kuşdemir (2016) was not accessible to the authors of the current paper.

## Supplementary Materials

The following are available online at https://www.mdpi.com/article/10.3390/jintelligence12020014/s1, S1: Alternative CFA models, Table S1: Fit indices for the original and alternative latent factor models of the anxiety questionnaires, Sample A.

## Appendix A

**Table A1:** Questions by questionnaire.

Code	Question	Mean	Factor Loading
*Children’s Math Anxiety Questionnaire Revised (CMAQ-R; Ramirez et al. 2016).*			
MA1 *	Look at this graph. It shows the number of cans the teacher has collected for a competition. How would you feel when you were asked how many cans Mary had collected?	1.64	-
MA2	How would you feel when you are in the arithmetic lesson and your teacher will learn something new?	1.77	0.576
MA3 *	How would you feel when having to solve this problem: How much money does Annie have when she has 2 euros in one hand and 4 eurocents in the other?	1.39	-
MA4 *	How would you feel if your teacher would ask you for the number of squares in this image?	1.38	-
MA5	Look at the clock. How would you feel if you were asked what time it will be in 20 minutes?	1.90	0.652
MA6	How would you feel if you had to sit down and start your math homework?	1.78	0.624
MA7 *	How do you feel when finding out if you have enough money to buy candy and a can of soda?	1.74	-
MA8	How do you feel when your teacher explains how to solve arithmetic problems?	1.84	0.592
MA9 *	How do you feel when solving 27 + 15?	1.49	-
MA10	How do you feel when you take a big test in the arithmetic lesson?	2.92	0.801
MA11	How do you feel when you take your arithmetic book and see all the numbers in it?	1.82	0.648
MA12	How do you feel when you are in the arithmetic lesson and you don’t understand something?	2.54	0.744
MA13 *	How would you feel when having to solve this problem: You score 15 points, your friend scores 8 points. How many points did you score more than your friend?	1.32	-
MA14 *	How would you feel when having to solve this problem: There are 13 ducks in the water. There are 6 ducks at the grass. How many ducks are there in total?	1.21	-
MA15	How do you feel when the teacher asks you to solve a calculation exercise on the blackboard?	2.31	0.767
MA16	How would you feel when you have to solve 34–17?	1.61	0.510
*Children’s Reading Anxiety Questionnaire (CRAQ)*			
RA1	Look at this text. How would you feel if you were asked to read it?	1.66	0.655
RA2	How do you feel during the language lesson when your teacher says that you will have to read a new text?	1.61	0.676
RA3	How would you feel if you had to start a new assignment reading comprehensively?	1.95	0.681
RA4	How would you feel if your teacher asks you questions about what you have read?	2.32	0.681
RA5 *	How would you feel reading a shopping list to check whether everything is in your cart?	1.47	-
RA6	How do you feel when to sit down and start your homework, which consists of reading a new book?	1.72	0.622
RA7	How do you feel when you have to look something up in a dictionary?	1.61	0.480
RA8	How do you feel when your teacher explains the text reading comprehensively?	1.60	0.544
RA9	How do you feel when you have to read the word ‘cholesterol’ out loud?	1.90	0.721
RA10	How do you feel when you have to take a reading test in the language lesson?	2.10	0.658
RA11	How do you feel when you take your reading writing/book and see all the texts and words in it?	1.65	0.623
RA12	How do you feel when you are in the language class and do not understand a word?	2.04	0.577
RA13	How would you feel if you had to read as many words as possible in 1 min?	2.65	0.761
RA14	How would you feel if you had to read this text?	1.52	0.588
RA15	How do you feel when the teacher asks you to read out loud a text for the classroom?	1.87	0.751
RA16	How would you feel if you had to read the word ‘aluminum’ out loud?	1.51	0.500
*Subtest Self-confidence during tests (SVL; Smits and Vorst 2008)* ^1^			
TA1 *	Optimistic after completing a test. (Reversed)	2.29	-
TA2	Fear of forgetting something	1.84	0.461
TA3	Fear of tests	2.39	0.449
TA4 *	Confident after completing a test. (Reversed)	2.51	-
TA5	Worries after announcement of a test	2.00	0.587
TA6	Calm and concentrated during a test (Reversed)	2.29	0.425
TA7 *	Nervous when test results are published.	1.54	-
TA8 *	Optimistic when handing in completed test. (Reversed)	2.50	-
TA9	Self-confidence before test-taking. (Reversed)	2.27	0.449
TA10 *	Nervous when test results are published.	1.57	-
TA11	Nervous in spite of good preparation.	1.89	0.505
TA12 *	Confident when teacher asks questions. (Reversed)	2.41	-
TA13	Worries about test grades.	1.88	0.524
TA14 *	Confident when prepared for a test. (Reversed)	2.56	-
TA15	Feeling nervous after announcement of a test.	1.69	0.417
TA16	Self-confidence about test results. (Reversed)	2.33	0.373

Note. ^1^ Due to copyright regulations, only a description of each question is provided. * Item was deleted from the factor model to optimize model fit.

## Appendix B

SEM plots for models including math performance measures.
