# Supplementary material for: Anxiety about Mathematics and Reading in Preadolescents Is Domain-Specific
Source: J Intell. 2024 Jan 29;12(2):14. doi: 10.3390/jintelligence12020014 (PMC10890043; doi:10.3390/jintelligence12020014)
Supplement: Supplementary file 1 [file jintelligence-12-00014-s001.zip › jintelligence-2591964-supplementary.pdf]

Alternative CFA models

Two types of alternative models were tested using confirmatory factor analysis (CFA). First, feelings may be different in a test context than when working on academic tasks without anything being at stake. Whereas the TA questionnaire exclusively asks about the test context, questions in the MA and RA questionnaires either refer to a test context or to a context in which the student performs a math or reading task without being tested. Second, anxiety reports may be impacted by the specific anxiety component that is being queried. Whereas items of the TA scale concern either affect or cognition, the MA and RA questionnaires only concern affect.

Context-specific Models

The first context-specific model is in line with the option that the anxieties are part of one overarching construct and had two factors: "Content" and "Test". Content-related questions of the reading anxiety scale and the math anxiety scale were forced to load on the factor "Content", whereas all test-related questions of these scales and all questions of the test anxiety scale loaded on the factor "Test".

The second context-specific model started from the option that the anxieties were separate and had five factors: "Content Reading Anxiety" (content-related questions of the reading anxiety scale loaded on this factor), "Content Math Anxiety" (content-related questions of the math anxiety scale loaded on this factor), "Test Reading Anxiety" (test-related questions of the reading anxiety scale loaded on this factor), "Test Math Anxiety" (test-related questions of the math anxiety scale loaded on this factor), and "Test anxiety" (all questions of the test anxiety scale loaded on this factor).

The fit measures are in Table S1. The fit measures of the 2-factor model were unsatisfactory and worse than that of the 5-factor model but the covariance matrix of the latent variables in the 5-factor model was not positive definite. We conclude that including context-specificity did not improve the factor models.

Models in which Anxiety Components were taken into Account

The first model was based on the assumption that the anxieties are part of one overarching construct and had two factors: "Affect" and "Cognition". Affect-related questions of the test anxiety scale and all questions of the reading anxiety scale and the math anxiety scale were forced to load on factor "Affect", whereas the cognition-related questions of the test anxiety scale loaded on the factor "Cognition".

The second model was based on the assumption that the anxieties were separate while taking the anxiety components into account. This resulted in four factors: "Affect Reading Anxiety" (all questions of the reading anxiety scale loaded on this factor), "Affect Math Anxiety" (all questions of the math anxiety scale loaded on this factor), "Affect Test Anxiety" (all affect-related questions of the test anxiety scale loaded on this factor), and "Cognition Test Anxiety" (cognition-related questions of the test anxiety scale loaded on this factor).

The fit measures are again in Table S1. The fit measures of the model with separate factors for the anxieties were better than those of the model with factors "Affect" and "Cognition" only. Also, the fit measures were very comparable to the original model with three factors. Following the procedure described for the original model, we first deleted items with factor loadings lower than 0.3, and next items with factor loadings lower than 0.4. However, fit measures CFI and TLI remained unsatisfactory.

Table S1. *Fit indices for the original and alternative latent factor models of the anxiety questionnaires, Sample A.*

Model	χ^2	*df*	χ^2/ds	RMSEA	SRMR	CFI	TLI
Models for original research question							
1-factor (original)	2895.00	1080	2.68	0.08	0.09	0.64	0.63
3-factor (original)	1970.35	1077	1.83	0.05	0.06	0.83	0.82
3-factor (original), items deleted with loadings ≤ .3[b]	1849.80	986	1.88	0.06	0.06	0.83	0.83
3-factor (original), items deleted with loadings ≤ .4[b]	847.94	492	1.72	0.05	0.05	0.91	0.90
Context-specific models							
2-factor: Test & Test-free	2797.39	1079	2.59	0.08	0.09	0.66	0.65
5-factor: Test.MA, Test.RA, TA, Test-free.MA, Test-free.RA[a]	1946.71	1070	1.81	0.05	0.06	0.84	0.83
Component-specific models							
2-factor: Anxiety & Cognition	2705.81	1079	2.51	0.07	0.08	0.68	0.67
4-factor: MA, RA, TA.Anxiety, TA.Cognition	1883.37	1074	1.75	0.05	0.06	0.85	0.84
4-factor, items deleted with loadings < .3[b]	1817.37	1028	1.77	0.05	0.06	0.85	0.84
4-factor, items deleted with loadings ≤ .4[b]	1158.53	623	1.86	0.05	0.06	0.88	0.87

Note. [a] Covariance matrix of latent variables is not positive definite. [b] Items with factor loadings ≤ .3/.4 in the previously estimated model were deleted. TA: test anxiety; MA: math anxiety; RA: reading anxiety.
